# R-Smad Competition Controls Activin Receptor Output in *Drosophila*

**DOI:** 10.1371/journal.pone.0036548

**Published:** 2012-05-01

**Authors:** Aidan J. Peterson, Philip A. Jensen, MaryJane Shimell, Ray Stefancsik, Ranjula Wijayatonge, Rachel Herder, Laurel A. Raftery, Michael B. O'Connor

**Affiliations:** 1 Department of Genetics, Cell Biology, and Development, University of Minnesota, Minneapolis, Minnesota, United States of America; 2 Cutaneous Biology Research Center, Massachusetts General Hospital/Harvard Medical School, Charlestown, Massachusetts, United States of America; 3 School of Life Sciences, University of Nevada Las Vegas, Las Vegas, Nevada, United States of America; Technische Universität Dresden, Germany

## Abstract

Animals use TGF-β superfamily signal transduction pathways during development and tissue maintenance. The superfamily has traditionally been divided into TGF-β/Activin and BMP branches based on relationships between ligands, receptors, and R-Smads. Several previous reports have shown that, in cell culture systems, “BMP-specific” Smads can be phosphorylated in response to TGF-β/Activin pathway activation. Using *Drosophila* cell culture as well as *in vivo* assays, we find that Baboon, the *Drosophila* TGF-β/Activin-specific Type I receptor, can phosphorylate Mad, the BMP-specific R-Smad, in addition to its normal substrate, dSmad2. The Baboon-Mad activation appears direct because it occurs in the absence of canonical BMP Type I receptors. Wing phenotypes generated by Baboon gain-of-function require Mad, and are partially suppressed by over-expression of dSmad2. In the larval wing disc, activated Baboon cell-autonomously causes C-terminal Mad phosphorylation, but only when endogenous dSmad2 protein is depleted. The Baboon-Mad relationship is thus controlled by dSmad2 levels. Elevated P-Mad is seen in several tissues of dSmad2 protein-null mutant larvae, and these levels are normalized in *dSmad2*; *baboon* double mutants, indicating that the cross-talk reaction and Smad competition occur with endogenous levels of signaling components *in vivo*. In addition, we find that high levels of Activin signaling cause substantial turnover in dSmad2 protein, providing a potential cross-pathway signal-switching mechanism. We propose that the dual activity of TGF-β/Activin receptors is an ancient feature, and we discuss several ways this activity can modulate TGF-β signaling output.

## Introduction

TGF-β superfamily signaling controls a multitude of metazoan cellular behaviors. Much of the signaling activity can be explained by the canonical Smad pathway, wherein extracellular ligands direct formation of active receptor complexes that phosphorylate and activate R-Smad transcription factors [1]. Multiple levels of regulation are known, ranging from extracellular ligand processing to diverse post-translational modification of Smad proteins [2]–[3]. In addition, a number of Smad-independent signaling pathways have also been demonstrated for various receptors and in different cell types [4].

Two functional subfamilies in TGF-β signaling are generally recognized based on genetic phenotypes and molecular studies: the TGF-β/Activin branch and the BMP branch. Phylogenetic analysis indicates that the TGF-β and BMP branches are present in all metazoans [5], suggesting that a robust TGF-β signaling network is an ancient feature of multicellular animals. The key difference between the two branches is that they activate different classes of R-Smads and thus induce different transcriptional responses in target cells. The TGF-β/Activin ligands work through Smad2/3 (dSmad2 in *Drosophila*) and BMP ligands work through Smad1/5/8 (Mad in *Drosophila*). These selective outputs are orchestrated by the Type I receptors, which bind to specific extracellular ligands and phosphorylate specific subsets of R-Smads.

Although genetic and molecular biology studies have consistently indicated that TGF-β/Activin and BMP signals use different R-Smads, there have been several reports of activation of BMP R-Smads by TGF-β ligands. This cross-talk activity was observed in several types of mammalian cells, but conflicting mechanisms were put forth on how this is achieved. Goumans et al. [6] described phosphorylation of BMP R-Smads upon TGF-β ligand exposure in human endothelial cells, and they concluded that TGF-β and BMP Type I receptors are both required for the reaction. Another report suggested that TGF-β phosphorylation of BMP R-Smads in human epithelial cells is accomplished by a BMP receptor [7]. In that model, the receptor signaling complex again includes two Type I receptors from different signaling branches: a TGF-β/Activin receptor that both acts as a scaffold for the ligand and phosphorylates TGF-β/Activin R-Smads, and a BMP receptor that phosphorylates BMP R-Smads. However, two other studies demonstrated that a human TGF-β/Activin receptor can phosphorylate BMP R-Smads directly, eliminating any role for a BMP receptor in some cell types [8]–[9]. Activin-to-BMP cross-talk in *Drosophila* cell culture has also been reported [10], but the receptor directly responsible for the Activin-stimulated phosphorylation of Mad, the mechanisms by which this phosphorylation are regulated, and whether such cross-pathway signaling occurs *in vivo* at endogenous levels of the various signaling components are not known.

In this report, we further explore these issues using both cell culture and *in vivo* methods. We conclude that phosphorylation of the *Drosophila* BMP R-Smad, Mad, by Baboon, encoded by the only Type I TGF-β/Activin family receptor, is direct and is an evolutionarily conserved signaling event. Furthermore, we find that competition between R-Smads has a profound influence on Baboon-to-Mad phosphorylation activity and that R-Smad pathway switching can be promoted by loss of dSmad2.

## Results

### Activation of Baboon by mutation or by ligand stimulation leads to phosphorylation of both Mad and dSmad2

**Figure 1:**
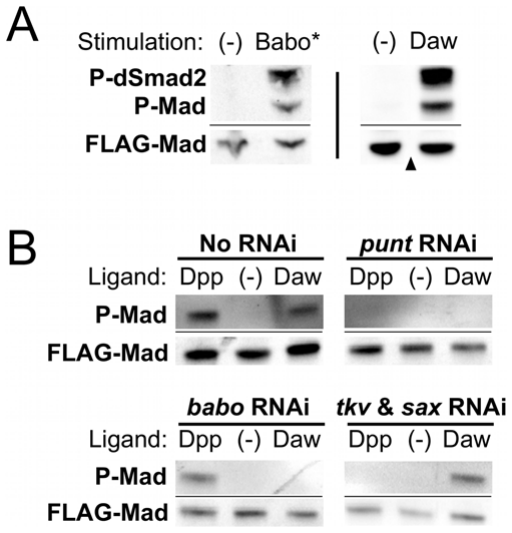
Stimulation of the Baboon receptor leads to phosphorylation of both R-Smads independently of BMP Type I receptors. S2 cells were transiently transfected with FLAG-tagged Smad expression constructs and analyzed by Western blot for C-terminal phosphorylation (P-dSmad2 and P-Mad). The FLAG-Mad band is shown as a loading control (FLAG-dSmad2 is not a useful loading control because of signaling-induced degradation as described later in the text). For all Western blot figures, a thin horizontal line indicates different infrared channels from the same blot. Co-expression of a constitutively active form of Baboon (Babo*) led to phosphorylation of dSmad2 and Mad (A, left blot). Exposure of cells expressing endogenous Baboon to the Dawdle ligand (Daw) had the same effect (A, right blot). RNAi treatment was used to determine receptor requirement for ligand activity (B). Controls confirmed that Dpp ligand treatment caused Mad phosphorylation independently of Baboon (B, left half). Dpp activity required the Punt Type II receptor and the Tkv and Sax BMP Type I receptors (B, right half). In contrast, Daw signaling to Mad required Baboon and Punt, but not Tkv and Sax.

In order to define the molecular nature of cross-talk, we employed a cell-based signaling assay that we have used to study other aspects of TGF-β signaling [11]. Expression of a constitutively active form of Baboon (Babo*) in *Drosophila* S2 cells resulted in phosphorylation of both the Activin and BMP R-Smads, dSmad2 and Mad (Figure 1A). To ensure that this phosphorylation of Mad is a property of the Baboon receptor itself and is not due to an artifact of the activating mutation in Babo*, we exposed S2 cells to the Activin-like ligand Dawdle (Daw), which signals through Baboon [12]. These cells, which express only endogenous Baboon, also responded to Daw by robustly phosphorylating both dSmad2 and Mad (Figure 1A). These results demonstrate that an Activin ligand can stimulate the BMP pathway in *Drosophila* S2 cells.

### Mad phosphorylation by Baboon is independent of BMP Type I receptors

As described above, a key mechanistic question is whether Baboon can directly phosphorylate Mad or if the traditional BMP receptors are involved. We addressed this point by conducting the signaling assay in cells lacking specific TGF-β-family receptors due to experimental RNA interference (RNAi). Similar to data shown in Figure 1A, control cells whose receptors were not targeted by RNAi accumulated phosphorylated Mad in response to the BMP-type ligand Decapentaplegic (Dpp) or the Activin-type ligand Daw (Figure 1B). Reducing expression of Punt, the only Type II receptor expressed in S2 cells, by RNAi eliminated the response to both ligands (Figure 1B). Cells lacking Baboon due to RNAi depletion still phosphorylated Mad in response to Dpp, but not in response to Daw (Figure 1B). In contrast, cells with both BMP Type I receptors Saxophone and Thickveins knocked down by RNAi still phosphorylated Mad upon exposure to Daw, even though they had no response to Dpp (Figure 1B). The abolishment of one or both ligand responses in each condition demonstrates the effectiveness of the various RNAi treatments. Additionally, the *Saxophone* and *Thickveins* RNAi treatments were validated for their ability to essentially eliminate expression of these proteins (see Figure S1). These results indicate that 1) BMP Type I receptors are neither necessary nor sufficient for Activin-induced phosphorylation of Mad in S2 cells, and 2) Baboon is the lone *Drosophila* Type I receptor that is necessary and sufficient for Activin-to-BMP cross-talk.

### Cross-talk is a conserved property of TGF-β receptor intracellular domains

**Figure 2:**
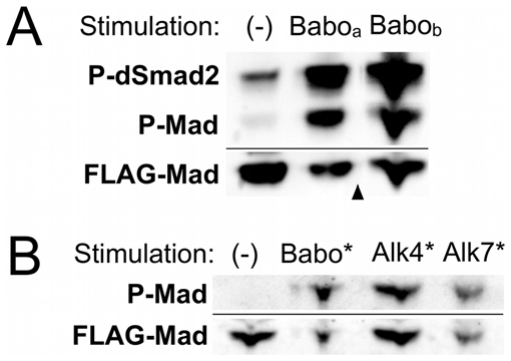
All isoforms of Baboon and mammalian Activin receptors can initiate cross-talk. (A) Overexpression of wildtype Babo_a_ or Babo_b_ in S2 cells led to phosphorylation of dSmad2 and Mad. In this experiment there was a detectable background level of P-dSmad2 well below the Babo-induced levels. Arrowhead indicates that the displayed image contains two portions of one blot. (B) Constitutively active versions of mammalian Activin receptors Alk4 and Alk7 (Alk4* and Alk7*), like Babo*, induce phosphorylation of *Drosophila* Mad in S2 cells.

Recent work has uncovered functional differences among isoforms of the sole Activin receptor in *Drosophila.* The three isoforms have divergent extracellular domains and identical kinase domains, and only one isoform, Babo_c_, is activated by the Daw ligand in S2 cells [12]. We wondered if the isoform differences might influence the ability to phosphorylate Mad, perhaps by directing different receptor complexes to form via extracellular interactions. We tested whether the other isoforms of Baboon, Babo_a_ and Babo_b_, were also able to stimulate cross-talk. When over-expressed in S2 cells, both of these isoforms displayed activity in the absence of exogenous ligand, and induced phosphorylation of both dSmad2 and Mad (Figure 2A). This result is consistent with studies suggesting that the kinase domain's L45 loop–a portion of the protein identical in Baboon isoforms–is primarily responsible for substrate recognition [13]. Extracellular differences among isoforms do not control cross-talk activity, supporting the simplest model of direct interaction between Mad and the kinase domain of Baboon.

Many reports have demonstrated that members of the same TGF-β-family pathway are interchangeable across species. For example, expression of a human BMP ligand can rescue mutation of a *Drosophila* BMP ligand *in vivo* [14]. We were curious if trans-species cross-talk was also possible. We tested whether activated TGF-β/Activin Type I receptors from mammals could phosphorylate the *Drosophila* BMP R-Smad. When transfected into S2 cells, the constitutively active forms of mammalian Activin receptors Alk4 and Alk7, like Babo*, could induce phosphorylation of both dSmad2 and Mad (Figure 2B and not shown). This interaction highlights the strong evolutionary conservation of the described cross-talk phenomenon.

### An *in vivo* Baboon gain-of-function phenotype is primarily mediated through Mad and not dSmad2

**Figure 3:**
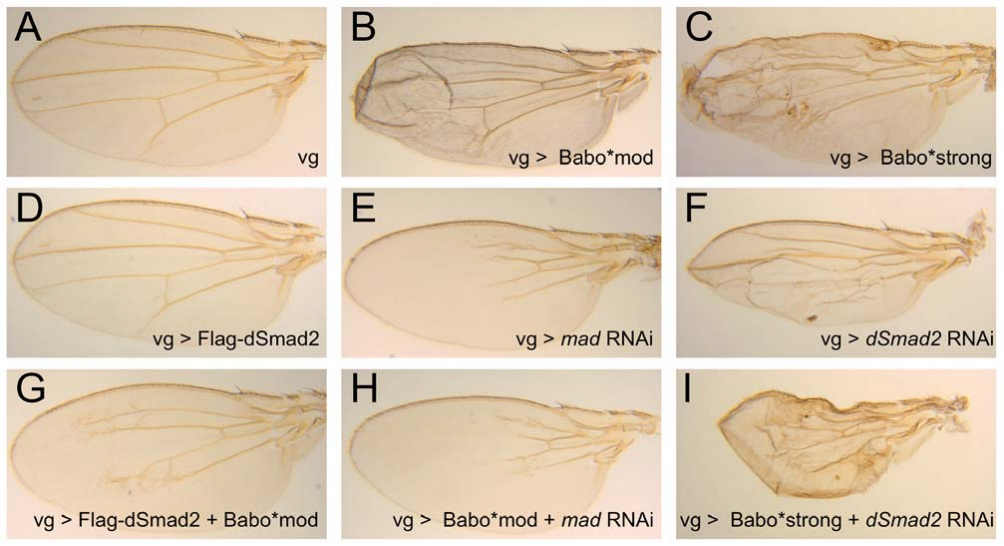
Excessive Baboon signaling perturbs wing development in a Mad-dependent manner. *vestigial-*GAL4 (*vg*) was used to express combinations of Baboon, dSmad2, and RNAi for *mad* and *dSmad2.* Normal wing development (A; one copy of *vg*-GAL4) was disrupted by Babo* expression (B, C; Babo*mod and Babo*strong are UAS insertions with varying activity as characterized in other assays). The Babo* phenotype was abrogated by simultaneous *mad* RNAi (H) and resembled *mad* RNAi alone (E). In contrast, the crumpling defect of Babo* wings was enhanced in conjunction with *dSmad2* RNAi (I) and was more severe than *dSmad2* RNAi alone (F). Overexpression of FLAG-dSmad2 did not affect wing formation (D), but partially rescued the Babo* overexpression phenotype, producing normal sized flat wings with residual peripheral vein defects (G). For each genotype, a representative wing is shown out of 4–7 wings photographed. Within a genotype there was only slight variation in appearance, except that 3/6 dSmad2 RNAi wings had vein defects and a blister (shown) and 3/6 had vein defects without a blister (not shown).

Given the demonstrated ability of Baboon to phosphorylate Mad and dSmad2 in cell culture, we looked for evidence that Baboon can signal through Mad *in vivo*. Previous studies have shown that moderate expression of Babo* in developing wings can generate enlarged but normally patterned wings [15]. We found that Babo* expression in the imaginal wing using the *vg*-GAL4 driver led to visible adult wing defects (Figure 3B, C). To determine whether activation of Mad or dSmad2 caused the phenotype, we used RNAi to remove one R-Smad at a time in wings expressing Babo*. *vg*-GAL4 driving *mad* RNAi alone produced a small, flat wing with missing veins (Figure 3E), whereas the effect of *dSmad2* RNAi knockdown alone was a moderately blistered wing (Figure 3F). When *mad* was removed in the presence of Babo*, the blistered and crumpled Babo* wing phenotype was suppressed (Figure 3H), and the resulting wings resembled those expressing *mad* RNAi alone. In contrast, removal of *dSmad2* by RNAi did not suppress the wrinkling phenotype induced by Babo*; in fact, the phenotype became more severe. The *dSmad2* RNAi, Babo* wings were more wrinkled and shrunken than wings where either construct was expressed alone (Figure 3I), suggesting that in the absence of dSmad2 more Mad is phosphorylated, perhaps because of less competition between dSmad2 and Mad for Baboon binding.

In support of this concept, we found that over-expression of dSmad2 suppressed the blistered wing phenotype of Babo* (Figure 3G), whereas dSmad2 over-expression alone did not affect wing size or vein formation (Figure 3D). This result rules out a general influence of dSmad2 on Mad activity and instead indicates that dSmad2 dosage specifically modifies the substrate choice of Baboon.

### dSmad2 directly influences Baboon's phosphorylation of Mad

Competition between dSmad2 and Mad for interaction with Baboon is a straightforward model. We carried out additional tests in cell culture and *in vivo* to confirm that the dSmad2 protein concentration affects Mad activation. As one test of the model of competitive binding between R-Smads, we manipulated levels of dSmad2, Baboon's hypothetically preferred substrate, in the S2 cell signaling assay.

**Figure 4:**
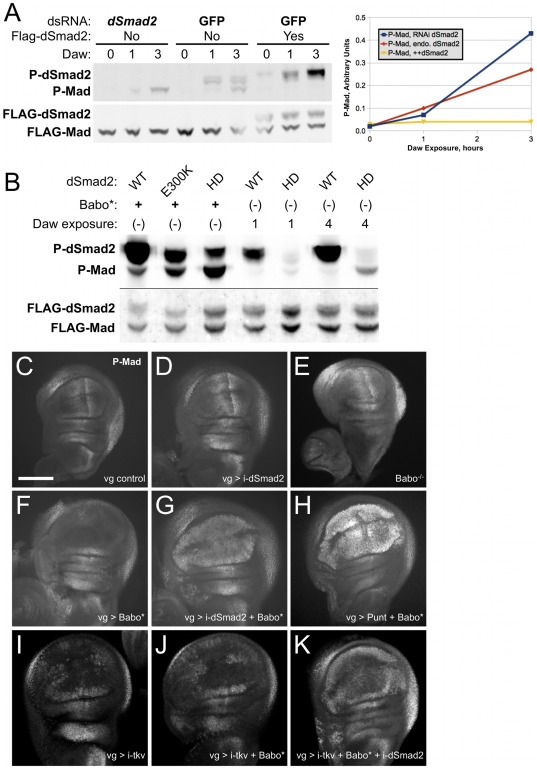
Baboon phosphorylation of Mad is inhibited by dSmad2. (A) Phospho-Smad accumulation upon exposure to Daw ligand. S2 cells transfected with Flag-Mad were treated with dsRNA for *dSmad2* or GFP, and transfected with Flag-dSmad2 as indicated. Phospho-Smad and Flag signals were assayed on samples before Daw exposure and after 1 and 3 hours of incubation. Note that there is a gel artifact affecting the appearance of the Flag bands in several lanes. Quantified P-Mad band intensities are plotted to illustrate that accumulation of P-Mad depends on the level of dSmad2. The experiment was repeated with several batches of reporter cells, and the relative signals for the 3 hour time point were always observed in the same order. The 1 hour time points were near background detection and are less reliable due to noise. (B) Mutated dSmad2 proteins with varying phosphorylation efficiency modulate the rate of P-Mad accumulation. Babo* stimulation revealed the steady-state levels of P-dSmad2 and P-Mad. Daw exposure for 1 or 4 hours showed the difference in response to short-term signaling between the WT and HD forms of dSmad2. In both conditions, P-Mad levels were inversely correlated to P-dSmad2 levels. (C–K) Wing imaginal discs from third instar larvae were stained to detect P-Mad and imaged by confocal microscopy. For each condition at least three discs were imaged, and a representative Maximal Intensity Projection encompassing the wing blade is shown (6 sections @ 3 micron interval for C,D,F–H; all sections @ 3 micron interval for E; 5 sections @ 2 micron interval for I–K). Anterior is to the left and the scale bar shown in panel C applies to C–K. The normal P-Mad staining pattern is shown for *vg*-GAL4 alone (C; scale bar = 100 microns). Expression of a UAS-*dSmad2* RNAi construct did not alter the P-Mad pattern or the shape of the wing disc (D). Wing discs from *babo^fd4/fd4^* homozygotes showed nearly normal pMad gradient (E). Expression of Babo* altered P-Mad staining, obliterating the normal gradient in the pouch (F). Note the normal P-Mad staining outside of the pouch where *vg*-Gal4 is not expressed. Babo* and *dSmad2* RNAi together generated ectopic P-Mad in the entire wing pouch (G). Providing Babo* with additional Punt also produced ectopic P-Mad (H). *tkv* RNAi prevented P-Mad accumulation in the middle of the wing pouch (I), and addition of Babo* did not counteract this P-Mad pattern (J). Additional knockdown of *dSmad2* led to ectopic P-Mad (K), which paralleled the results without *tkv* RNAi. Data in panels C, D, F, and G were from the same experiment and were stained in parallel. Panel H is from a different experiment, but the pouch signals can be compared to the others because the endogenous P-Mad along the posterior margin has similar staining. Samples in panels I–K were stained and processed in parallel.

As noted above, Babo* expression or prolonged Daw stimulation led to phosphorylation of Mad in S2 cells. Steady-state levels of P-Mad or P-dSmad2 after prolonged Babo* expression were similar whether Mad or dSmad2 were expressed individually or together (data not shown). As a more sensitive assay, we performed time-course experiments using Daw ligand to observe the initial kinetics of substrate phosphorylation. When Flag-dSmad2 was overexpressed, P-dSmad2 accumulated steadily over a several hour exposure to Daw (Figure 4A, right lanes). Endogenous P-dSmad2 was also detected upon Daw treatment, but the accumulation was not linear. The rate of P-Mad accumulation varied with the expression level of dSmad2. When Flag-Mad was the only over-expressed substrate, P-Mad was detected after 1 hour and continued to rise during 3 hours of ligand exposure (Figure 4A, middle lanes). This accumulation was modestly enhanced when endogenous dSmad2 was eliminated by RNAi (Figure 4A, left lanes). In the other direction, over-expression of Flag-dSmad2 suppressed the accumulation of P-Mad such that it was not detectable above background after 3 hours of Daw exposure (Figure 4A, right lanes). Quantified P-Mad signals are plotted in Figure 4A. This result implicates dSmad2 protein in controlling the rate of Mad phosphorylation by Baboon in S2 cells.

We considered several possibilities for how this might occur. First, in vertebrate systems, the Activin/TGF-β R-Smads are assisted in their presentation to a receptor by SARA (Smad Anchor for Receptor Activation) [16], an endosome-associated protein that potentially could provide a competitive edge to dSmad2 over Mad for phosphorylation by Baboon. However, RNAi knockdown of SARA in S2 cells had no affect on the dSmad2/Mad phosphorylation ratio (see Figure S2A), suggesting that differential presentation of dSmad2 to Baboon by SARA is not a likely mechanism for substrate choice. Another possibility is that molecular motors contribute to substrate preference by discriminately presenting one Smad to the receptor. In *Xenopus*, one report suggests that efficient Smad2 phosphorylation requires kinesin motors, presumably to help traffic Smad2 to the receptor [17]. We treated cells with colcemid to depolymerize microtubules and block potential transport mechanisms, but we did not observe a change in the relative phosphorylation of dSmad2 and Mad (see Figure S2B).

Since we found no evidence for differential presentation to receptors, we decided to test more directly the direct-binding/competition model by altering the receptor interaction interface of dSmad2 to make it more similar to Mad. We reasoned that if there was direct competition between these two R-Smads for binding to Baboon, then Baboon would phosphorylate both substrates at similar rates if they had similar interaction interfaces. Previous studies comparing vertebrate TGF-β/Activin Smad2/3 and BMP Smad1/5/8 identified two residues in the L3 loop of the MH2 domain as key for targeting different Smads to the L45 loop of either TGF-β/Activin or BMP receptors [18]. We changed the R446 and T449 residues of dSmad2 to H and D, respectively, to give dSmad2 a Mad-like L3 loop. We also made a variant of dSmad2 that harbors the E300K substitution in the MH2 domain found in the *dSmad2^MB388^* allele [19].

We compared the relative phosphorylation levels of wildtype (WT) and mutant forms of dSmad2 versus Mad in response to Baboon. In the experiment shown in Figure 4B, the steady-state P-dSmad2(WT) signal was greater than the P-Mad signal, and the E300K and HD variants had reduced P-dSmad2. Importantly, the P-Mad signal increased as the P-dSmad2 signal decreased, which is consistent with the substrate competition model. This behavior was confirmed in a Daw time-course experiment for dSmad2(WT) and dSmad2(HD). During the first hours of signaling, phosphorylation of the HD variant was dramatically reduced compared to WT. P-Mad accumulation, modest over this time range when forced to compete with dSmad2(WT), was enhanced when pitted against the less effective dSmad2(HD) protein (Figure 4B).

### dSmad2 restricts Baboon-Mad cross-talk activity *in vivo*

These signaling assays in S2 cells demonstrate that the Baboon kinase can use Mad as a substrate and that dSmad2 levels modulate the phosphorylation of Mad by Baboon. We next sought to determine if these two phenomena occur *in vivo*. We chose the wing disc as a platform to manipulate Baboon because the normal P-Mad pattern is well characterized and, as described below, seems to be independent of canonical dSmad2-Baboon signaling. Wing discs from third-instar larvae have a well-defined pattern of P-Mad that critically depends on the Dpp ligand and the BMP Type I and Type II receptors Tkv and Punt, respectively. *baboon* is expressed in the wing disc, but *baboon* mutants display a normal P-Mad pattern ([15] and Figure 4E). Similarly, *dSmad2* is expressed in discs, but removal of dSmad2 in the wing pouch by RNAi did not significantly alter the P-Mad pattern (Figure 4D, compare to 4C). The lack of an aberrant P-Mad pattern after reduction of either Baboon or dSmad2 is consistent with a lack of appreciable cross-talk activity in the larval wing disc at this developmental time under endogenous conditions.

Against this backdrop, we manipulated Baboon signaling in wing discs and used P-Mad staining patterns as an indicator of activity. When expressed in the L3 wing disc blade using a *vg*-GAL4 driver, constitutively active Babo* altered the P-Mad pattern. Curiously, the P-Mad signal was reduced in the wing blade, disrupting the normal gradient pattern (Figure 4F). This resembled a loss-of-function condition for Thickveins (compare to Figure 4I) that is likely caused by titration of Punt away from BMP signaling complexes. Indeed, when Punt was co-expressed with Babo*, P-Mad was detected throughout the *vg*-GAL4 pouch region (Figure 4H). The Punt expression line used for this experiment did not induce ectopic P-Mad in the wing pouch by itself (not shown). It is likely that Babo*-Punt complexes are active towards dSmad2, but we could not directly verify this because no P-dSmad2 immunohistochemistry (IHC) detection assay is available. However, we were able to show that dSmad2 impacts the output of Babo* by simultaneously expressing Babo* and knocking down endogenous *dSmad2*. This combination produced strong ectopic P-Mad throughout the *vg*-Gal4 expression domain (Figure 4G). Once again, we interpret these data as reflecting a competition between dSmad2 and Mad proteins for binding to and activation by Baboon. Reinforcing the result seen in S2 cells, this *in vivo* scenario implicates dSmad2 as the preferred substrate for Baboon since endogenous dSmad2 protein can prevent over-expressed Baboon from phosphorylating Mad.

We also tested if Babo* phosphorylation of Mad can occur independently of the relevant BMP Type I receptor in the wing disc. Simultaneous Babo* expression and *dSmad2* RNAi led to ectopic P-Mad even with effective knockdown of *tkv* (Figure 4K). In concordance with our cell culture studies, this result indicates direct Baboon to Mad cross-talk *in vivo*.

### Dosage effects at endogenous levels: P-Mad levels increase in some tissues of dSmad2 protein-null animals

**Figure 5:**
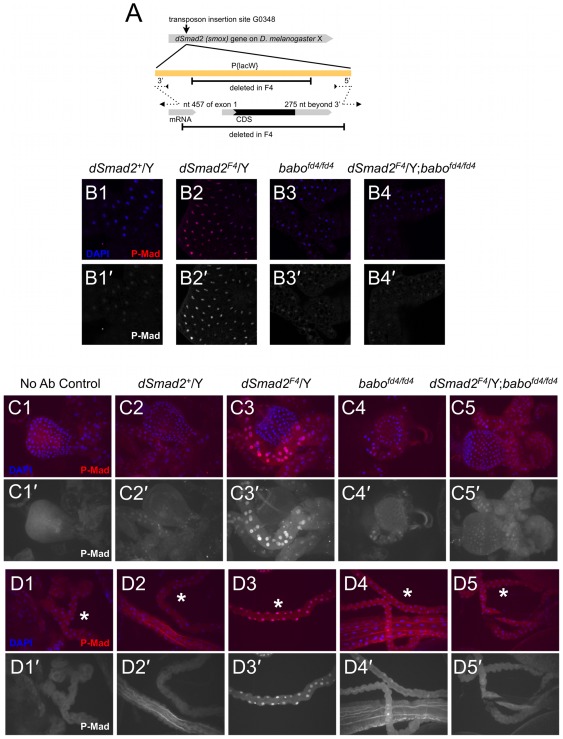
P-Mad elevation in *dSmad2* null mutant tissues depends on *baboon*. The schematic depicts the location of the l(X)G0348 *P* element insertion in relation to the *dSmad2* locus (A). The F4 excision product removed the entire coding region of *dSmad2* and portions of the *P*-element. The genomic breakpoints are indicated above the *dSmad2* mRNA; they were determined by sequencing PCR products, indicated by dotted lines. (B–D) P-Mad was detected by IHC of fixed larval tissues from several genotypes. For each image, a merged DAPI (blue) and P-Mad (red) panel is displayed above the isolated P-Mad channel. (B) Single confocal sections of P-Mad staining in the fat body. Under the staining conditions employed, endogenous nuclear P-Mad in a control fat body was barely detected (B1), but was increased in a *dSmad2* null mutant animal (B2). *Baboon* single mutants and *dSmad2*; *baboon* double mutants had normal P-Mad staining (B3,4). (C, D) P-Mad staining at two representative positions along the digestive tract. Images are Maximal Intensity Projections of 3 micron interval confocal sections through the entire sample. The P-Mad primary antibody was omitted from “No Ab Control” samples to convey any background staining and auto-fluorescence in the red channel. (C) In the gastric caeca near the proventriculus, *dSmad2* mutants showed elevated P-Mad (C3) compared to wildtype control males (C2). *baboon* single mutants and *dSmad2*; *baboon* double mutants showed wildtype levels (C4 and C5). Distal Malpighian tubule staining (lumpy tubes marked with asterisks) showed the same pattern, with the *dSmad2* mutant displaying the strongest P-Mad staining (D3 compared to D2, D4 and D5).

Given the observation that the presence of dSmad2 in the wing disc profoundly alters the activity of exogenous Babo*, we asked if a similar effect is observed with endogenous levels of TGF-β signaling components. To do this, we required a protein-null condition to preclude competition between Mad and dSmad2 binding to Baboon. The only available dSmad2 mutant allele, *dSmad2^MB388^*, harbors a mis-sense mutation near the beginning of the MH2 domain. This mutation does not prevent interaction with Baboon, as the corresponding E300K protein was robustly phosphorylated in S2 cells by Babo* (Figure 4B). To generate a protein null allele, we excised P(G0348), a *white*-marked *P* element located approximately 400 nt downstream of the *dSmad2* transcriptional start, and screened for *white^−^* lethal progeny (see Materials and Methods). Among several candidates, one allele, *dSmad2^F4^*, was a deletion of the entire coding sequence (Figure 5A). We next surveyed P-Mad levels by IHC in various tissues derived from *dSmad2* mutant larvae. We observed that many, but not all, tissues displayed greater P-Mad staining in the *dSmad2* mutant compared to control animals reared under the same conditions. For example, nuclear P-Mad in the fat body was weakly detected in control animals, but markedly elevated in *dSmad2^F4^* mutants (Figure 5B1 vs FB2). A similar increase was seen at various positions along the gut tract: P-Mad staining was elevated in the gastric caeca, the midgut/hindgut junction, and Malpighian tubules compared to equivalently stained control larvae (examples in Figure 5C and 5D, and summary in Figure S3).

We next tested the influence of the Baboon receptor on P-Mad levels in the fat body and gut. In contrast to *dSmad2* mutants, *baboon* mutant animals had normal P-Mad staining (Figure 5B3, 5C4 and 5D4). The low level of nuclear P-Mad seen in wildtype and *baboon*-mutant gut tissue is presumably due to canonical BMP-family signaling through Thickveins and Saxophone receptors. We next performed an epistasis test in which we assayed P-Mad levels in a *dSmad2*; *babo* double mutant. We reasoned that if the ectopic P-Mad in a dSmad2 protein-null mutant is due to enhanced phosphorylation of Mad by Baboon, then it should be suppressed in the double mutant. We observed this result in all tissues examined (Figure 5B4, 5C5 and 5D5, and Figure S3). Thus, Baboon function is required to produce the elevated P-Mad levels observed in dSmad2 deficient tissues. To explain this phenomenon we conclude simply that Baboon activity is directed towards Mad when dSmad2 is not present *in vivo*.

### Baboon activation causes bulk degradation of dSmad2

**Figure 6:**
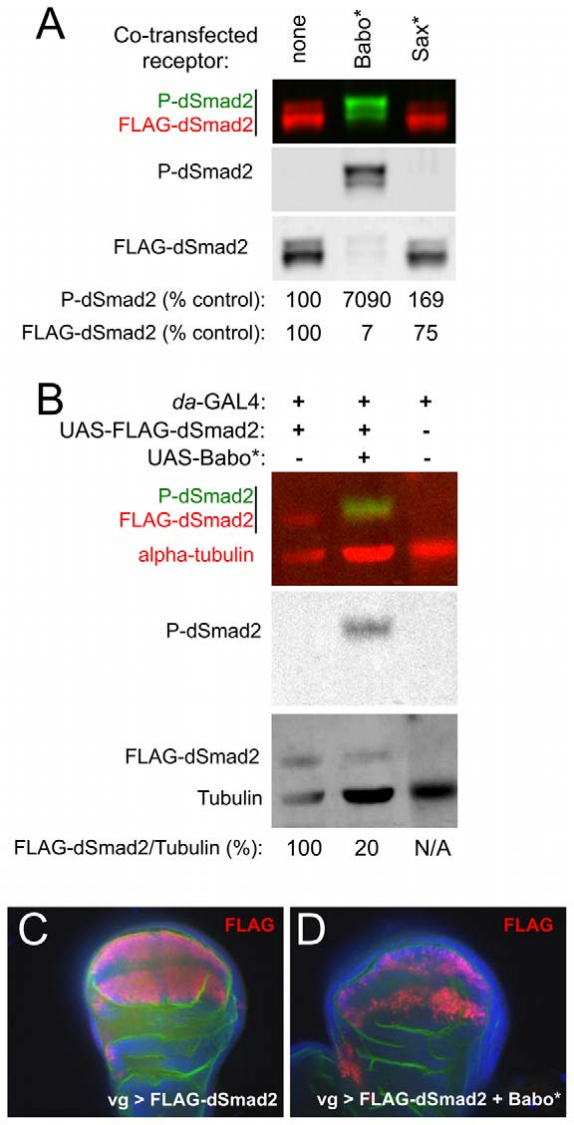
Hyperactive Baboon signaling leads to dSmad2 destabilization. The steady-state level of transfected dSmad2 in S2 cells was significantly reduced when Babo* was co-expressed (A). Quantified band intensities are displayed as a percentage of the control for each channel. Note that the P-dSmad2 level was greatly increased but the total dSmad2 level was reduced in the presence of Babo*, causing a greater than 1000-fold increase in the ratio of P-dSmad2 to total Flag-dSmad2. This effect was specific to Babo*, as inclusion of activated Saxophone (Sax*) neither stimulated P-dSmad2 nor significantly decreased the FLAG-dSmad2 signal (A, right lane). FLAG-dSmad2 was expressed in embryos using the *da*-GAL4 driver, either alone or with Babo* (B). Total exogenous dSmad2 and P-dSmad2 was detected by Western blot and the amount of FLAG-dSmad2 was normalized to endogenous alpha-tubulin. (C, D) *vg*-GAL4 was used to express Flag-dSmad2 alone (C) or with Babo* (D) in the wing disc. FLAG antibody IHC was used to gauge dSmad2 levels (red), and DAPI (blue) and aPKC IHC (green) are shown as counter-stains. Each image is a single confocal plane through the wing blade, and represents at least three discs stained per genotype.

During our experiments in S2 cells, we observed that over-expression of activated Baboon significantly lowered the level of co-over-expressed dSmad2 protein. In these experiments S2 cells were transfected with actin promoter expression constructs and were assayed several days later. Thus the dSmad2 protein level detected by Western blot represents the steady-state level, which reflects the production and degradation rates. Protein production rates should be constant in this system, so changes in the steady-state level of dSmad2 can be interpreted as changes in the degradation rate. By this measure, the degradation rate of dSmad2 increased about 5- to10-fold when Babo* was present (Figure 6A).

We next tested this behavior in several *in vivo* situations to determine the generality of the bulk degradation of dSmad2 upon Baboon activation. Western blot detection of dSmad2 from embryo extracts showed that co-expression of Babo* lowered the dSmad2 level about 5-fold, compared to dSmad2 expression alone (Figure 6B). A similar reduction of exogenously expressed dSmad2 was seen in the wing blade upon expression of Babo* (compare Figure 6D to 6C). These results confirm that Baboon stimulation lowers dSmad2 stability *in vivo*. Below we consider how such degradation could alter the output of Baboon signaling in the context of cross-talk.

## Discussion

### Comparison with cross-pathway signaling in other systems

In this report we present experimental results showing that the Baboon receptor can directly phosphorylate Mad in cell culture and *in vivo*, and that this cross-talk activity is tightly controlled by the availability of dSmad2. These findings extend the initial report describing canonical signaling between Baboon and dSmad2 where dSmad2, but not Mad, was shown to be a substrate of Baboon in mammalian cells [15]. There are several possible reasons why Baboon-Mad activity is observed in *Drosophila* cells but was missed in the initial report. First, it is possible that Mad binding to Baboon may be too weak or transient to be detected by immunoprecipitation. Alternatively, endogenous Smad2/3 may have blocked the interaction in heterologous cell systems, or species-specific co-factors may facilitate Mad-Baboon binding.

The strong functional conservation of TGF-β superfamily proteins prompts a comparison of our results in *Drosophila* with studies describing cross-pathway signaling in mammalian systems. The limited number of pathway members and the efficacy of RNAi in *Drosophila* enabled us to distinguish between competing models presented for mammalian epithelial cells. With regard to the key mechanistic question of whether TGF-β Type I receptors can directly phosphorylate the BMP R-Smads, our observation of Mad phosphorylation in the absence of BMP Type I receptors (Figure 1) is inconsistent with the model of heteromeric TGF-β/BMP Type I receptor complexes proposed by Daly et al. [7], but is consistent with the model of direct phosphorylation of BMP R-Smads by TGF-β/Activin receptors proposed by Liu et al. [8]. It is possible that both types of mechanisms exist, but are differentially utilized depending on the ligands and receptors present in the particular tissue being examined. Even in *Drosophila*, we note that direct action of Baboon on Mad does not preclude the possibility that mixed receptors form active signaling complexes in some situations. Mixed receptor complexes have been detected in *Drosophila* cell culture under over-expression conditions, but their functionality is unknown [20]. A similar point can be made regarding mixed Smad oligomers detected in mammalian epithelial cells by Daly et al. [7]. In our adult wing assay, we found that the Babo* phenotype depended primarily on Mad and that dSmad2 was not required. If the wing development defect was caused by the activity of a complex containing both P-Mad and P-dSmad2, then removal of either one should have blocked the Babo* phenotype. Again, this observation does not argue against the formation or activity of mixed R-Smads complexes in some contexts, but shows that productive signaling by cross-pathway phosphorylation can take place independently of such complexes.

One key observation in this report concerning the mechanism of cross-pathway signal regulation is that the degree to which it occurs, both in cell culture and *in vivo*, appears to be regulated by competition between the R-Smads, likely for receptor binding. Further work is required to determine how general this mechanism might be. Epithelial cell culture models showed that cross-talk is important for the TGF-β-induced migratory switch [8]. Our results in the larval wing disc and gut represent the first examples of cross-talk *in vivo*, and we expect that additional examples will be found in various animals and tissues. With regard to developmental studies, other systems should be evaluated to see if loss-of-function mutations of Smad2/3 orthologs lead to increased signaling through BMP R-Smads. Additionally, several human diseases have been attributed to mutations in TGF-β components [21]. A cautionary implication of our work is that mutations in the TGF-β branch may have unanticipated loss- or gain-of-function influences on the BMP branch.

Curiously, TGF-β/Activin Type I receptors appear to have gained or retained cross-phosphorylation activity throughout evolution, but BMP Type I receptors do not appear to have reciprocal activity. We have never seen phosphorylation of dSmad2 by activated *Drosophila* Type I BMP receptors (Figure 6A and data not shown), or in response to various ligands. Likewise, no phosphorylation of Smad2/3 by BMP receptors has been reported in vertebrate cells. Why is there this distinction? The growing number of complete genome sequences has allowed phylogenetic reconstruction of the evolution of TGF-β signaling [5]. Apparently all metazoans, even the simple placazoan [22], have a complex TGF-β network containing both TGF-β/Activin and BMP subfamilies. It is tempting to speculate that a single receptor with dual Smad targets could have played a transitional role in the expansion of the signaling network. The all-or-none nature of the TGF-β-superfamily network (both BMPs and TGF-β/Activins present, or none) in extant organisms, however, does not provide support for this idea. What it does support is the possibility that relationships between core pathway proteins stabilized hundreds of millions of years ago. If the dual-kinase activity of Baboon orthologs has been available to all metazoans during the radiation of animal forms, we would expect this activity to be deployed by different animals and different cell types in diverse ways. We are currently investigating several phenotypes that differ between *baboon* and *dSmad2* mutants to determine which depend on Babo-Mad cross-talk and which might depend on other forms of Smad-independent signaling [23].

### Smad2 degradation as a signaling switch

The different responses of adult wings and larval imaginal tissue upon Baboon activation (Figures 3 and 4) illustrate that TGF-β pathway wiring and output can vary with developmental context. Given the relationship between Babo and dSmad2, the cross-talk activity can be viewed two ways. From one perspective, the response to loss of dSmad2 depends on the level of Baboon signaling: only cells with Baboon activity can produce P-Mad by cross-talk. From the other perspective, the response of wildtype cells to Baboon stimulation depends on dSmad2 levels: efficient cross-talk will only occur in the absence of dSmad2.

**Figure 7:**
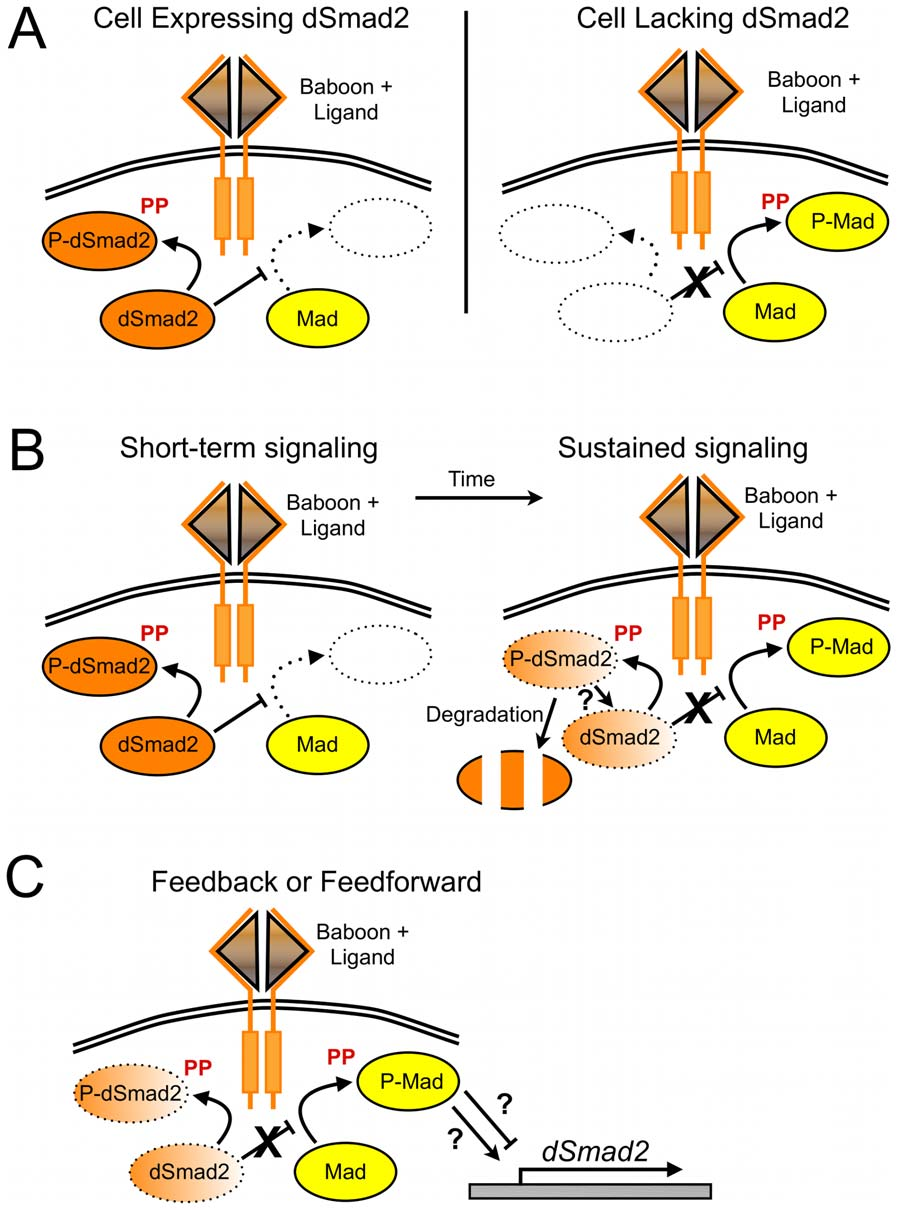
Models of signaling outputs generated by dual kinase activity of the Baboon receptor. Given the potential of Baboon to phosphorylate and activate dSmad2 and Mad, we present several configurations of Baboon, dSmad2 and Mad interaction that would generate different signaling outputs. In each schematic, only the most relevant pathway components are drawn. The first model represents a logic gate, where the ability of Baboon to phosphorylate Mad is strictly controlled by the expression of dSmad2 in that cell (A). Another model incorporates the observed degradation of dSmad2 in response to Baboon signaling (B). The final model (C) incorporates feed-back or feed-forward of Mad activity through *dSmad2* regulation. If P-Mad positively regulated *dSmad2* this would prevent the complete loss of dSmad2. Conversely, if P-Mad negatively regulated *dSmad2* expression, this would enforce the switch from Baboon signaling through dSmad2 towards Mad.

Given the dominant control that dSmad2 exerts on the ability of Baboon to phosphorylate Mad, the simplest model is that output of TGF-β ligand stimulus in a given cell depends on the expression level of dSmad2 (Figure 7A). Although dSmad2 is widely expressed, as are Smad2/3 proteins in other animals, different subsets of cells might express different ratios of R-Smads that could influence the signaling output.

Our observation that Baboon activity can lower the overall level of dSmad2 protein offers an additional regulatory possibility, where the TGF-β/Activin signal itself can influence its response. In the substrate-switch model, a cell exposed to a prolonged Activin signal would eventually degrade enough of its dSmad2 pool to allow Baboon signaling through Mad (Figure 7B). It is not known which tissues, if any, require Baboon-to-Mad signaling in normal developmental contexts. We have observed that in some cases, dSmad2 over-expression leads to *mad* loss-of-function phenotypes, which could represent disruption of a sensor incorporating dSmad2 concentration as an input and Mad activity as an output (unpublished observations). Proteosomal degradation of activated Smad proteins is a well documented mechanism of signal attenuation [24], but the current observation of bulk degradation adds a new dimension because it can redirect receptor signaling. The mechanism of signal-dependent bulk dSmad2 degradation is unknown, and preliminary experiments do not support a simple ubiquitylation-proteosomal pathway (unpublished observations). Regardless of the molecular details, the observed reduction of total dSmad2 available for receptor competition is the key parameter in our competition model. Since activated R-Smad proteins can also be dephosphorylated to rejoin the pool of would-be substrates, the relative rates of recycling and bulk degradation would be predicted to influence the substrate switch to Mad.

What could be gained by the substrate switch? A general answer is that multiple interactions permit diverse regulatory schemes. For example, cross-talk would permit Mad signaling in a cell that is not exposed to BMP ligands or is otherwise not competent to transduce such signals. Other modes of signal integration are intriguing, such as the conditional formation of mixed R-Smad complexes observed by Daly et al. [7]. Another type of regulatory link that could contribute to the evolutionary maintenance of an integrated dSmad2-Babo-Mad triad is depicted in Figure 7C. If *dSmad2* transcription were a target for P-Mad, this would affect the substrate switch. In once case, Baboon signaling to Mad would be triggered by dSmad2 depletion and serve to up-regulate Smad2 to return balance to the system. On the other hand, if dSmad2 were down-regulated by P-Mad, this would stabilize the switch to the Baboon-Mad interaction. Additional studies are required to explore these intriguing possibilities.

## Materials and Methods

### Signaling assays in S2 cells

The S2 signaling assay has been described [12]. Briefly, conditioned media was collected from cells that expressed a construct encoding Dawdle. This media was mixed 1∶1 with cells that had been transfected with a combination of Smads [11] and resuspended in their own media. Conditioned media from cells that were mock transfected was used as a negative control; recombinant Dpp (R&D Systems) was added to conditioned media at a final concentration of 20 nM for BMP stimulation. Mutated forms of dSmad2 (E300K and HD = R446H+T449D) were generated by site-directed mutagenesis and the changes were verified by sequencing. Constructs encoding different isoforms of Baboon are described by Jensen et al. [12]. Expression vectors for Babo* and Sax* are described by Gesualdi and Haerry [10]. Mammalian Alk4* and Alk7* [25], [26] were cloned into the pAcPA vector for S2 cell expression.

RNAi treatment of S2 cells was as described [12], except that for Figure 4A dsRNA was added only once after transfection. Primers used to make PCR products for T7 transcription templates against *tkv*, *sax*, and *punt* are described by Shimmi and O'Connor [27] and GFP dsRNA was used as a negative control [28]. Additional primers for RNAi are as follows:

*dSmad2* forward–TAATACGACTCACTATAGGGAGAGGGCAAGTGGTCAGAGAA;
*dSmad2* reverse–TAATACGACTCACTATAGGGATGCCCACACTAAGCACACTC;
*babo* forward–TAATACGACTCACTATAGGGACCTGCGCTCCGGCTAATCTTCC;
*babo* reverse–TAATACGACTCACTATAGGGACGGATGATAGCCACAAACTCCC;
*dSARA* forward–TAATACGACTCACTATAGGGGTATCGTCCTCCGAATCGCTGAC;
*dSARA* reverse–TAATACGACTCACTATAGGGGATTTAGGGTTTGTGGTCGCTGGGG.

Cells for microtubule disruption tests were exposed to a final concentration of 2 µg/mL colcemid in their own media for 2 hours prior to signaling assays. Control samples were treated similarly, but without drug pre-treatment. After the signaling assay, cells were pelleted, resuspended in 1× sample buffer, and boiled. Samples were resolved on NuPAGE 4–12% Bis-Tris gels (Invitrogen) and transferred to PVDF or nitrocellulose (Figure 4A only) membrane after electrophoresis. Membranes were probed with primary antibodies against C-terminally phosphorylated Mad (1∶1000, gift from E. Leof), phosphorylated dSmad2 (1∶1000, Cell Signaling #3108), the FLAG epitope (1∶2000, Sigma (M2)), or Saxophone (1∶1000, Fabgennix). Membranes were exposed to secondary antibodies (Rockland (IRDye)) and imaged on the Odyssey Infrared Imaging system. To obtain fold change of degradation rates of FLAG-dSmad2 under steady-state conditions, we quantified the bands and calculated the ratio of unstimulated to Babo*-stimulated FLAG-dSmad2. This simple formula applies if production is constant and degradation is assumed to be a first order reaction.

### Fly lines

The *vg-*GAL4 driver (Bloomington stock #8229) was used to drive expression of several UAS constructs in the developing wing, and *da*-GAL4 (Bloomington stock #5460) was used to drive expression in the embryo. UAS-Babo* lines have been reported previously [15], as has the UAS-FLAG-dSmad2 overexpression line [29]. *dSmad2^MB388^* and *babo^fd4^* are described in Zheng et al. [19]. UAS-punt is described in Marqués et al. [30].

New RNAi lines were made using the pUAST-R57 vector (National Institute of Genetics, Japan) and included gene-specific sequences spanning bases 1264–1700 of the *mad* transcript (U10328; insertion line 6C3), 1469–1928 of the *dSmad2* transcript (AF101386; insertion line 25A3 or 3B3), and 926–1461 of the *tkv* transcript (NM_175975; recombinant line 38D2, 1A2). *P*-element-mediated insertions were made and mapped using standard techniques.

### Larval IHC and tissue handling

Tissues from wandering third-instar larvae were fixed in 3.7% formaldehyde in PBS for 25 minutes, washed in PBS-Triton (0.1%), treated with primary antibody overnight at 4°C, exposed to secondary antibodies (Alexa Fluor 488 or 568; 1∶200) at room temperature for two hours, and counterstained with DAPI. The P-Mad antibody (gift from E. Leof) was affinity purified against a peptide corresponding to the di-phosphorylated C-terminus of Mad. M2 monoclonal antibody (Sigma) was used to detect FLAG-tagged proteins. Anti-PKCζ (rabbit polyclonal, Santa Cruz) was used to visualize cell membranes for Figure 6. Confocal images were collected using a Zeiss Axiovert microscope with a CARV attachment, or with a Zeiss LSM710. The images in Figure 4C–H and 6C,D were collected using a 20×/0.75NA objective (CARV). The images in Figure 5C,D were collected using a 10×/0.45NA objective (CARV). The images in Figure 4I–K and Figure 5B were collected using a 20×/0.80 NA objective (LSM). Where indicated in Figure Legends, Maximum Intensity Projections were generated using Axiovision (CARV images) or ImageJ (LSM images) software.

Wings from adult females were dissected in ethanol and mounted in 1∶1 Canada balsam∶wintergreen oil under a coverslip for light microscopy using a 4×/0.16NA objective. All flies for wing and wing disc studies were reared on standard cornmeal medium at 25°C. Larvae for whole-animal P-Mad IHC were grown on yeast paste agar plates, and mutant animals were identified by the absence of GFP balancers. Embryo extracts were made by boiling dechorionated 6–20 hour old embryos in 2× reducing sample buffer, and the soluble fraction was used for Western blot analysis.

### Generation of a null *dSmad2* mutant

A new *dSmad2* allele was generated by imprecise excision of a *P* element from stock l(X)G0348 (Flybase allele *smox^G0348^*), which is located in the *dSmad2* 5′ UTR. *P*-element males carrying a duplication (Tp1;2 sn+ 72d) to provide viability were outcrossed, and male progeny lacking the visible eye color marker were recovered and tested for lethality. Lethal chromosomes were analyzed by PCR and sequencing to map the extent of deletion. The *dSmad2^F4^* deletion removed a portion of the 5′ UTR (break point at nucleotide 457 of sequence AF101386) and the entire coding region. The 3′ breakpoint was found to be 275 bp downstream of the *dSmad2* transcript and the deletion does not disrupt the neighboring gene. Several segments of the original *P* element were left behind (see Figure 5A). Hemizygous *dSmad2^F4^* males died as larvae or pupae. The *dSmad2^F4^* allele was fully rescued to viability and fertility by one copy of a small duplication covering *dSmad2* (either Dp(1;3)DC185 or Dp(1;3)DC186). An independent excision allele was generated in parallel from a *yellow*-marked *P*-element insertion (Flybase allele *smox^GG01120^*), using a slightly different strategy (details available upon request to LAR). The *dSmad2^C15^* allele from this screen was found to be an internal deletion of the transposable element. Like *dSmad2^F4^*, hemizygous *dSmad2^C15^* males died as larvae or pupae and were fully rescued by duplications overlapping *dSmad2*. We chose the *F4* allele for our mutant analysis because it is an unambiguous protein null.

## Supporting Information

Figure S1 **Efficient knockdown of** ***sax*** **and** ***tkv*** **in S2 cells.** (A) Cells were transfected with a Sax over-expression (O/E) construct with or without *sax* RNAi treatment. Western blotting for Sax showed that the RNAi treatment rendered Sax undetectable. (B) A similar test for Tkv showed approximately 98% reduction of overexpressed Tkv-FLAG upon RNAi as detected by FLAG Western blot. In both panels, the loading control is a FLAG cross-reactive band from the same gel lanes. Note that endogenous Sax and Tkv are not detected under these conditions.
(TIF)

Figure S2 **Relative phosphorylation of dSmad2 and Mad are unaffected by knockdown of** ***SARA*** **or disruption of microtubules.** (A) Smad phosphorylation upon Daw treatment in S2 cells without or with *dSARA* RNAi. As judged by the FLAG bands (red in merged image, and isolated in bottom slat), the RNAi samples had less Smad per lane, but the relative P-dSmad2 versus P-Mad ratios were similar between control and *dSARA* RNAi samples. (B) Colcemid treatment did not affect the preferential activation of dSmad2 by Baboon. In this particular experiment pMad stimulation by Daw was weak, but P-Mad was not increased in cells pre-treated with colcemid to depolymerize microtubules. This is in contrast to the increase in P-Mad caused by knockdown of dSmad2. Together these suggests that dSmad2 delivery to Baboon is not compromised upon microtubule disruption. In both panels, rDpp was included as a control for the ability of the cells to produce P-Mad.
(TIF)

Figure S3 **P-Mad elevation in** ***dSmad2*** **mutants requires** ***baboon*** **, and occurs in tissues that express a** ***dSmad2*** **reporter.** (A) A chart summarizing P-Mad IHC staining results in a panel of larval tissues. At least six animals were examined for control and *dSmad2* mutants, and three animals were examined for *babo* and *dSmad2*; *babo* mutants. Some gut sections were lost during staining; n.d. indicates that the tissue was not photographed for that genotype. (B) LacZ staining in G0348 heterozygous female larvae was used as a proxy for *dSmad2* expression because the *P*-element insertion into the *dSmad2* 5′ UTR contains a LacZ reporter. Control animals are shown to indicate very low nuclear background staining. LacZ was readily detected in several alimentary tissues and in the fat body. Staining was weak in the salivary gland, which is a tissue where pMad does not increase in the *dSmad2* null mutant. Displayed images are Maximum Intensity Projections of confocal sections collected at 3 micron intervals, and were processed in parallel.
(TIF)
