# Supplementary figures and images for: R-Smad Competition Controls Activin Receptor Output in Drosophila
Source: PLoS One. 2012 May 1;7(5):e36548. doi: 10.1371/journal.pone.0036548 (PMC3341346; doi:10.1371/journal.pone.0036548)

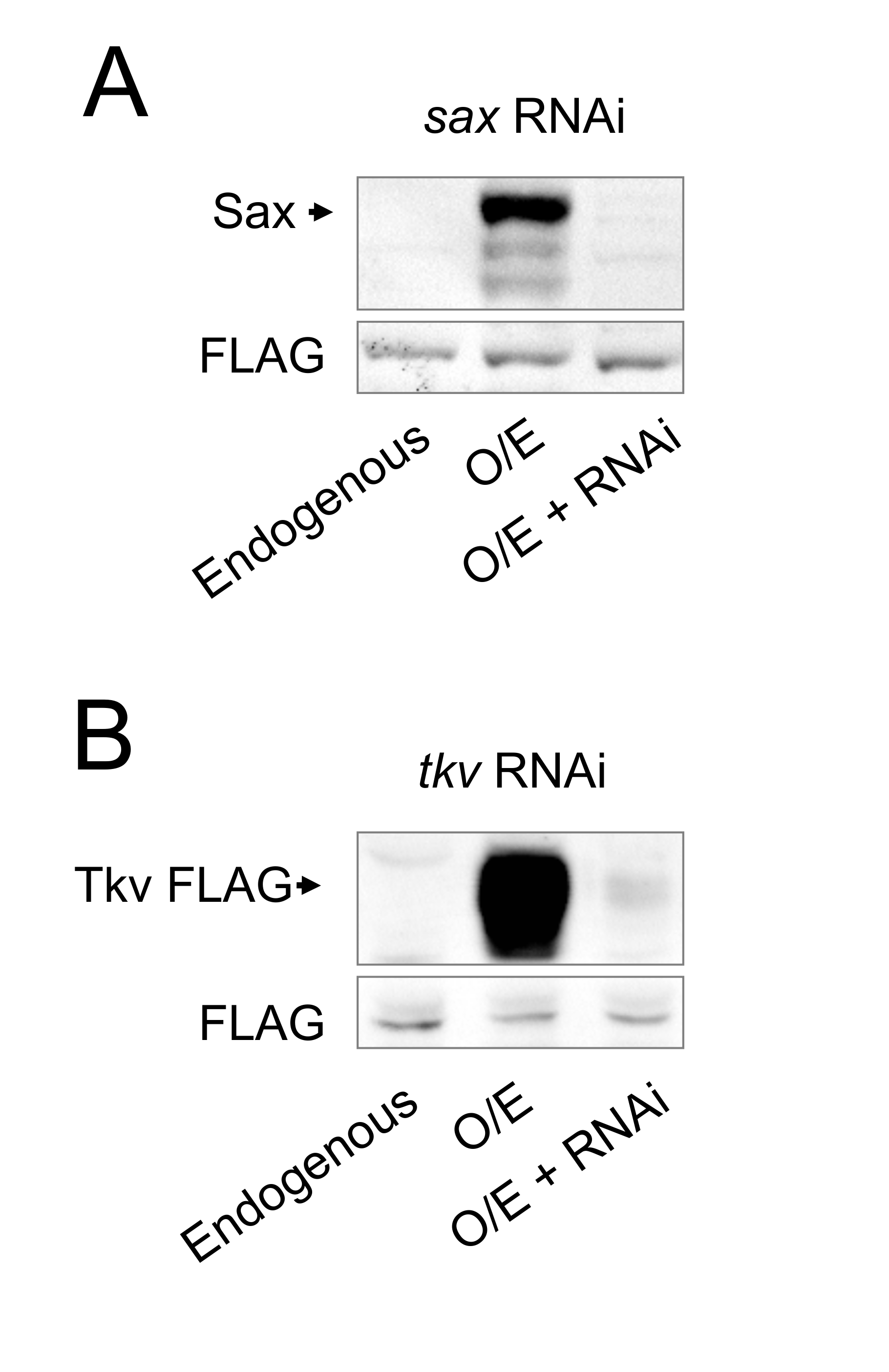

Supplement: Figure S1 — Efficient knockdown of sax and tkv in S2 cells. (A) Cells were transfected with a Sax over-expression (O/E) construct with or without sax RNAi treatment. Western blotting for Sax showed that the RNAi treatment rendered Sax undetectable. (B) A similar test for Tkv showed approximately 98% reduction of overexpressed Tkv-FLAG upon RNAi as detected by FLAG Western blot. In both panels, the loading control is a FLAG cross-reactive band from the same gel lanes. Note that endogenous Sax and Tkv are not detected under these conditions. (TIF) [file pone.0036548.s001.tif]

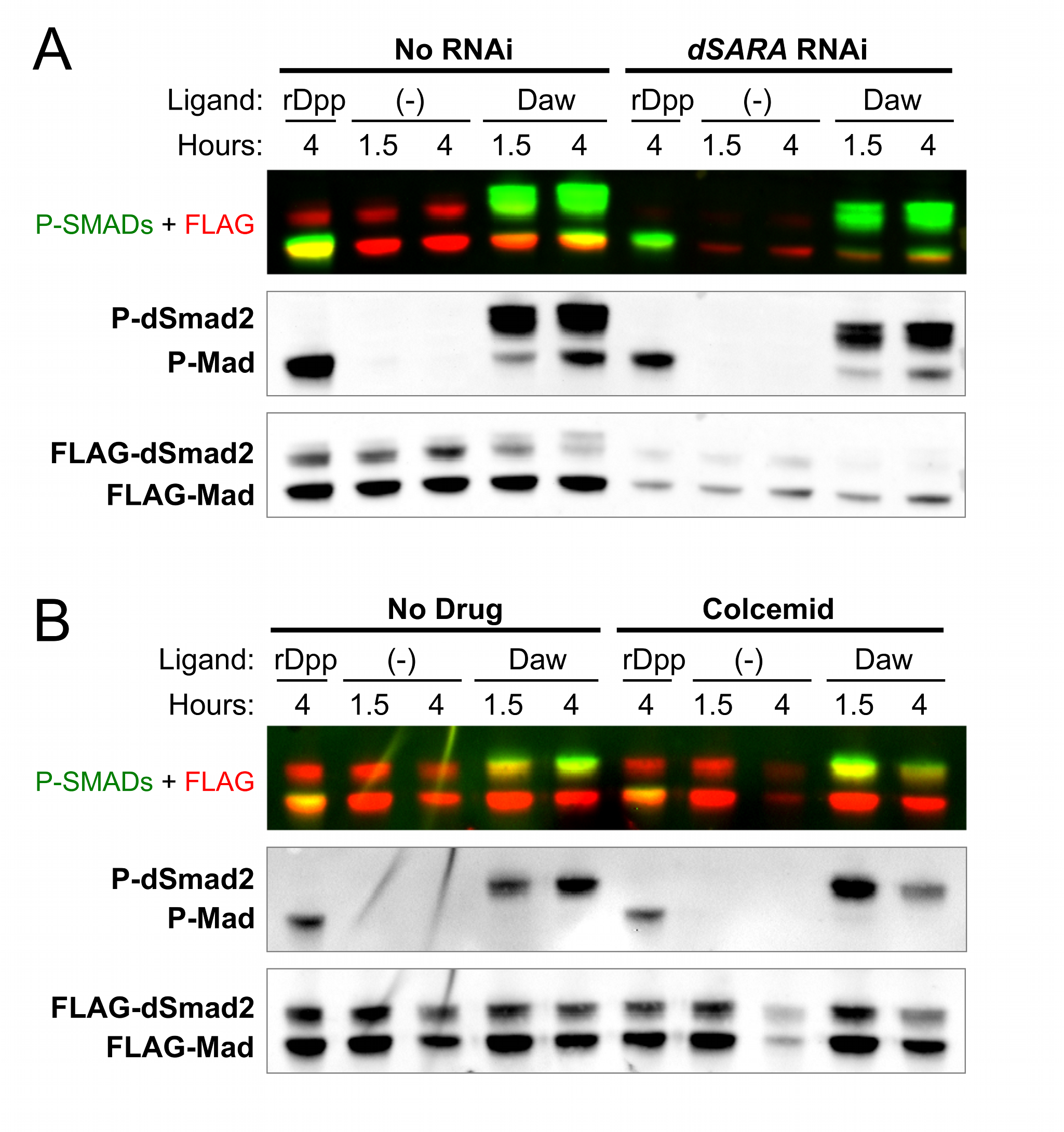

Supplement: Figure S2 — Relative phosphorylation of dSmad2 and Mad are unaffected by knockdown of SARA or disruption of microtubules. (A) Smad phosphorylation upon Daw treatment in S2 cells without or with dSARA RNAi. As judged by the FLAG bands (red in merged image, and isolated in bottom slat), the RNAi samples had less Smad per lane, but the relative P-dSmad2 versus P-Mad ratios were similar between control and dSARA RNAi samples. (B) Colcemid treatment did not affect the preferential activation of dSmad2 by Baboon. In this particular experiment pMad stimulation by Daw was weak, but P-Mad was not increased in cells pre-treated with colcemid to depolymerize microtubules. This is in contrast to the increase in P-Mad caused by knockdown of dSmad2. Together these suggests that dSmad2 delivery to Baboon is not compromised upon microtubule disruption. In both panels, rDpp was included as a control for the ability of the cells to produce P-Mad. (TIF) [file pone.0036548.s002.tif]

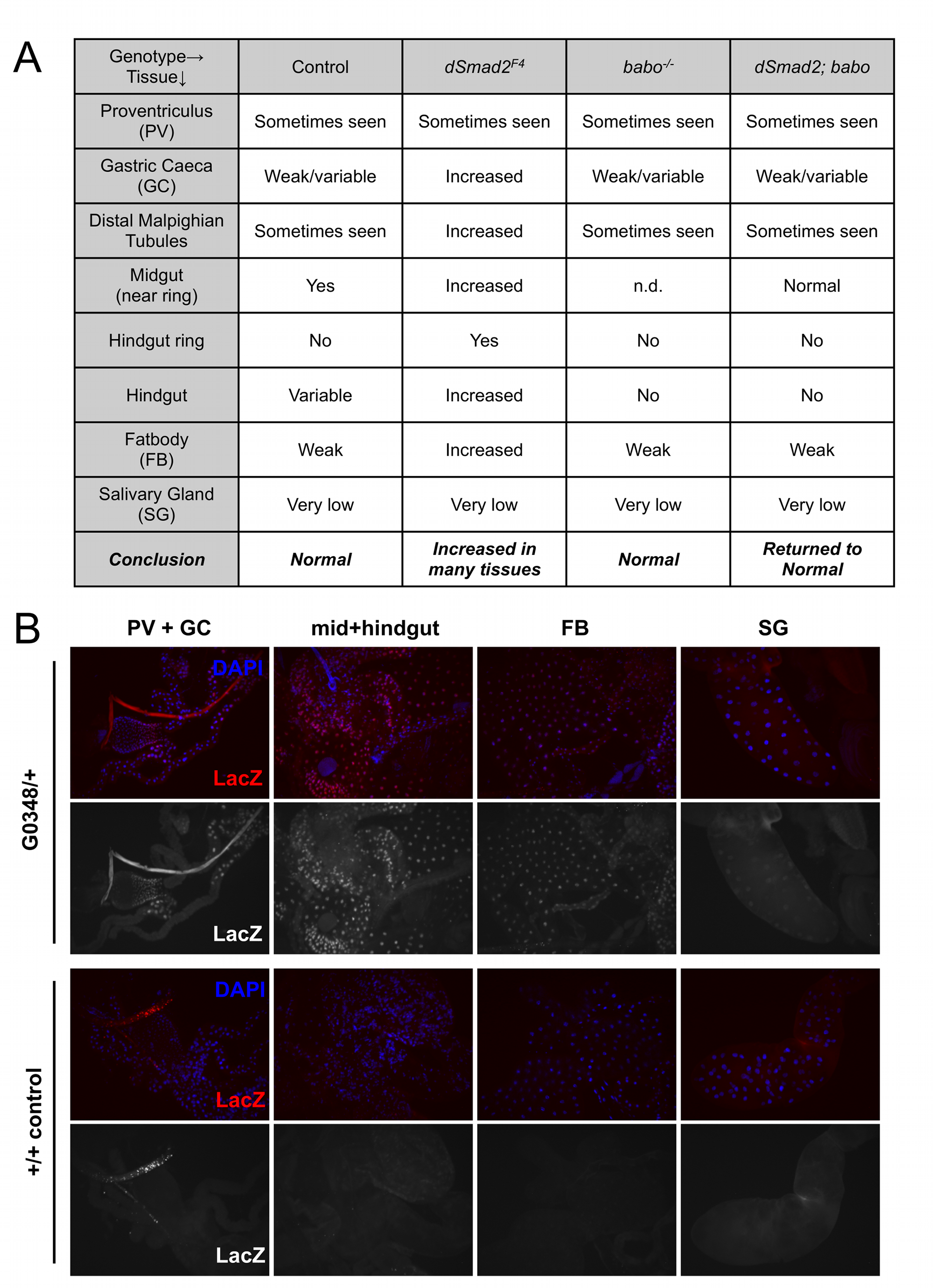

Supplement: Figure S3 — P-Mad elevation in dSmad2 mutants requires baboon , and occurs in tissues that express a dSmad2 reporter. (A) A chart summarizing P-Mad IHC staining results in a panel of larval tissues. At least six animals were examined for control and dSmad2 mutants, and three animals were examined for babo and dSmad2; babo mutants. Some gut sections were lost during staining; n.d. indicates that the tissue was not photographed for that genotype. (B) LacZ staining in G0348 heterozygous female larvae was used as a proxy for dSmad2 expression because the P-element insertion into the dSmad2 5′ UTR contains a LacZ reporter. Control animals are shown to indicate very low nuclear background staining. LacZ was readily detected in several alimentary tissues and in the fat body. Staining was weak in the salivary gland, which is a tissue where pMad does not increase in the dSmad2 null mutant. Displayed images are Maximum Intensity Projections of confocal sections collected at 3 micron intervals, and were processed in parallel. (TIF) [file pone.0036548.s003.tif]
